# miRNA expression profiles and molecular networks in resting and LPS-activated BV-2 microglia—Effect of cannabinoids

**DOI:** 10.1371/journal.pone.0212039

**Published:** 2019-02-11

**Authors:** Ana Juknat, Fuying Gao, Giovanni Coppola, Zvi Vogel, Ewa Kozela

**Affiliations:** 1 The Dr Miriam and Sheldon G. Adelson Center for the Biology of Addictive Diseases, Sackler Faculty of Medicine, Tel Aviv University, Tel Aviv, Israel; 2 Department of Neurobiology, Weizmann Institute of Science, Rehovot, Israel; 3 Departments of Psychiatry and Neurology, Semel Institute for Neuroscience and Human Behavior, David Geffen School of Medicine, University of California, Los Angeles, CA, United States of America; University of Florida, UNITED STATES

## Abstract

Mammalian microRNAs (miRNAs) play a critical role in modulating the response of immune cells to stimuli. Cannabinoids are known to exert beneficial actions such as neuroprotection and immunosuppressive activities. However, the underlying mechanisms which contribute to these effects are not fully understood. We previously reported that the psychoactive cannabinoid Δ^9^–tetrahydrocannabinol (THC) and the non-psychoactive cannabidiol (CBD) differ in their anti-inflammatory signaling pathways. Using lipopolysaccharide (LPS) to stimulate BV-2 microglial cells, we examined the role of cannabinoids on the expression of miRNAs. Expression was analyzed by performing deep sequencing, followed by Ingenuity Pathway Analysis to describe networks and intracellular pathways. miRNA sequencing analysis revealed that 31 miRNAs were differentially modulated by LPS and by cannabinoids treatments. In addition, we found that at the concentration tested, CBD has a greater effect than THC on the expression of most of the studied miRNAs. The results clearly link the effects of both LPS and cannabinoids to inflammatory signaling pathways. LPS upregulated the expression of pro-inflammatory miRNAs associated to Toll-like receptor (TLR) and NF-κB signaling, including miR-21, miR-146a and miR-155, whereas CBD inhibited LPS-stimulated expression of miR-146a and miR-155. In addition, CBD upregulated miR-34a, known to be involved in several pathways including Rb/E2f cell cycle and Notch-Dll1 signaling. Our results show that both CBD and THC reduced the LPS-upregulated Notch ligand Dll1 expression. MiR-155 and miR-34a are considered to be redox sensitive miRNAs, which regulate Nrf2-driven gene expression. Accordingly, we found that Nrf2-mediated expression of redox-dependent genes defines a Mox-like phenotype in CBD treated BV-2 cells. In summary, we have identified a specific repertoire of miRNAs that are regulated by cannabinoids, in resting (surveillant) and in LPS-activated microglia. The modulated miRNAs and their target genes are controlled by TLR, Nrf2 and Notch cross-talk signaling and are involved in immune response, cell cycle regulation as well as cellular stress and redox homeostasis.

## Introduction

MicroRNAs (miRNAs) are evolutionary conserved, endogenous non-coding, single-stranded small RNAs (19–23 nucleotides length) that regulate gene expression through post-transcriptional repression [1–2]. It has been demonstrated that miRNAs are involved in a wide variety of physiological processes, including cell proliferation and differentiation, development, apoptosis, metabolism, angiogenesis, immunity and homeostasis [3–5]. In contrast, dysregulated miRNA expression has been linked to a variety of acute and chronic diseases, including cancer, neurodegenerative disorders, autoimmunity, heart diseases, chronic viral infections as well as acute organ injury and ischemic stroke [6–9]. A number of miRNAs were found to be significantly upregulated in response to Toll-like receptor (TLR) ligands. These include miR-155, miR-146a and miR-21, which negatively regulate TLR signaling [4, 10–11]. TLRs initiate distinct signaling pathways depending on the adaptor molecules MyD88 and TRIF, leading to activation of the transcription factors NF-κB, AP-1, IRF3 and/or IRF7, which induce the production of pro-inflammatory cytokines and type I interferon (IFN) [12].

A growing body of evidence describes the role of miRNAs in macrophages and microglial cells under defined stimuli, which induce polarized phenotypes (M1/M2) [13–16]. Microglia are the resident macrophage-like cells of the central nervous system (CNS). Microglial cells actively scan their environment and contribute to the immune surveillance of the CNS [17]. There is increasing information about diversity as well as plasticity of microglial cells, from surveillance to phagocytic and activated states, showing a more complex phenotyping than the standard M1/M2. For example, Kadl et al., [18] defined the microglial phenotype Mox, characterized by the occurrence of Nrf2-mediated redox-regulated genes. The presence of this complex set of phenotypes leads to the heterogeneity of microglial cellular responses and to the wide range of space/time activation patterns of microglia [17,19].

Preparations derived from *Cannabis* (marijuana and hashish) are recognized nowadays as having many physiological effects and therapeutic applications [20–21]. The beneficial effects of marijuana and its active constituents, the cannabinoids, consist of suppression of nausea and vomiting, stimulation of appetite, relief of pain as well as amelioration of the unwanted symptoms of spasticity originated by multiple sclerosis (MS) [22]. In addition, cannabinoids are known to posses pro-apoptotic, neuroprotective and anti-tumor properties [23–25]. Amongst the different cannabinoids, Δ^9^-tetrahydrocannabinol (THC) and cannabidiol (CBD) are recognized for their potent immunosuppressive and anti-inflammatory properties [24, 26–34]. THC mediates its psychoactive activity by binding to the cannabinoid receptor CB1 and modulates its immune response primarily through activation of the cannabinoid receptor CB2. In contrast to THC, CBD shows little agonistic activity at CB1/CB2 receptors and is therefore devoid of the unwanted psychotropic effects, characteristic of THC action on CB1 receptor. Moreover, it has been reported that CBD displays some CB1/CB2 antagonistic activities [35]. More recently, Laprairie et al., [36–37] showed that CBD can serve as a non-competitive negative allosteric modulator of the CB1 receptor with the ability to inhibit its internalization.

CBD has been shown to exhibit many physiological effects, including anti-inflammatory, anti-oxidant and neuroprotective properties [28–32, 38–44]. These effects of CBD have been related to diverse non CB1/non CB2 mechanisms [43, 45–48].

By performing microarray analysis of genome-wide mRNA in microglial BV-2 cells, our group showed that CBD is associated to genes involved in the GCN2/eIF2α/p8/ATF4/Chop-Trib3 pathway. In addition, we reported that genes stimulated by CBD (mainly related to stress response and inflammation) are regulated via the (EpRE/ARE)-Nrf2/Atf4 system and the Nrf2/Hmox axis in LPS-treated microglial cells [31, 33].

The effect of THC on miRNA expression profiles has been studied in diverse inflammatory biological models by the group of Nagarkatti [49–52] and in simian immunodeficiency virus-infected macaques by the group of Molina [53–54]. However, to our knowledge, there are no reports showing how miRNAs regulate the CBD-associated immunosuppression effects.

In this work we studied the effects of the pro-inflammatory LPS, a powerful inducer of TLR4 signaling, on the expression of miRNAs in BV-2 microglial cells as well as the effects of the cannabinoids CBD and THC on the LPS-inflammatory response. Next generation sequencing was used to identify the differentially expressed miRNAs. Highly predictive miRNA target genes and their pathways were analyzed using bioinformatics computational tools. Our results show that CBD is a potent regulator of miRNA expression, in resting (surveillant) and in LPS-activated microglia. MiRNA target genes were found to be involved in immune response, cell growth and proliferation, cell death and survival as well as stress response and redox regulation. Identification of the CBD- and THC-modulated miRNAs and their related networks and pathways provide a molecular basis for understanding the effects of these cannabinoids on activated microglia.

## Materials and methods

### Materials

THC was obtained from the National Institute on Drug Abuse (Baltimore, MD, USA) and CBD was provided by THC Pharm GmbH (Frankfurt, Germany). Stock solutions of cannabinoids were prepared in ethanol and diluted in culture medium before experiments. Final ethanol concentration in the medium did not exceed 0.1%. Fetal calf serum (FCS), tissue culture reagents and medium were obtained from Biological Industries (Kibbutz Beit HaEmek, Israel). LPS from *Escherichia coli* (serotype 055:B5) was purchased from Sigma (St. Louis, MO, USA).

### Microglial cell culture

The immortalized murine BV-2 microglial cell line was kindly provided by Prof. E.J. Choi from Korea University (Seoul, Korea). BV-2 cells were grown in Dulbecco’s modified Eagle’s medium (DMEM) containing 4.5 g/l glucose, and supplemented with 5% FCS, penicillin (100 U/ml) and streptomycin (100 μg/ml) under a humidified 5% CO_2_ atmosphere at 37°C. Cells (1x10^6^ cells in 100 mm plates) were pretreated for 2h with either CBD or THC (both at 10 μM), followed by the addition of LPS (100 ng/ml) for another 4h. These concentrations and time points were chosen following our previous studies [28,31,33].

### Total RNA extraction

Total RNA (mRNA + miRNAs) was extracted using the NucleoSpin miRNA extraction kit (Macherey-Nagel; Düren, Germany) according to the manufacturer’s recommendation. Total RNA was quantified using the NanoDrop ND-1000 spectrophotometer (Thermo Scientific, Wilmington, DE), and the A260/230 and A260/280 ratios showing RNA purity, were examined. RNA quality was tested using the Eukaryote Total RNA Nano Assay, recorded with an Agilent 2100 Bioanalyzer (Agilent Technologies, Palo Alto, CA) and the RNA integrity number (RIN) was used as an RNA integrity parameter. Only samples with an RIN greater than 8 were used for further experiments and sequencing.

### miRNA sequencing

miRNA sequencing was performed at the Informatics Center for Neurogenetics and Neurogenomics core at the University of California Los Angeles (UCLA), USA. Eighteen sequencing libraries were prepared using Illumina TruSeq Small RNA Library Preparation Kit following manufacturer's protocol. All samples were multiplexed into a single pool in order to avoid batch effects [55] and sequenced using an Illumina HiSeq 2500 sequencer (Illumina, San Diego, CA) at 69bp-paired-end sequencing, yielding between 2.8 and 5.6 million reads per sample. Quality control (QC) was performed on base qualities and nucleotide composition of sequences. Reads were aligned to the miRBase mouse reference using the BWA read aligner, run allowing for a maximum of 10 mappings per read. Between 39 and 51% (average 46%) of the reads mapped uniquely to the mouse genome. Total counts of reads aligned to known microRNAs for mouse are used as the basis for quantification of gene expression. Differentially expressed genes were identified using the Bioconductor package edgeR [56] which were then considered and ranked based on adjusted p-values of ≤ 0.1. RNA seq data has been deposited within the Gene Expression Omnibus (GEO) public repository with the accession number GSE123262 (https://www.ncbi.nlm.nih.gov/geo/query/acc.cgi?acc=GSE123262).

### mRNA real time PCR (qPCR) analysis

The same RNA samples were used to test both mRNA and miRNA expressions by qPCR. For mRNA expression studies, cDNA was synthesized using the QuantiTect Reverse Transcription kit containing gDNA ‘wipe out’, (Qiagen AG, Basel, Switzerland) according to the manufacturer’s instructions. qPCR was carried out in the Rotor-Gene 3000 instrument, using Absolute Blue QPCR SYBR Green ROX mix containing the Thermo-Start DNA polymerase (Thermo Scientific, ABgene House, Epsom, Surrey, UK). Primer pairs for mRNA expression for selected transcripts were designed using the computer program Primer Quest, an online tool provided by Integrated DNA Technologies (http://test.idtdna.com/Primerquest/Home/Index). Wherever possible, designs with at least one of the primer sequences located on an intron-exon boundary were chosen, thus avoiding co-amplification of minor contaminating amounts of genomic DNA that could be present in the RNA samples. All primers were analyzed using the nucleotide program BLAST (http://blast.ncbi.nlm.nih.gov/Blast.cgi) to ensure primer specificity for the gene of interest. Designed primers were synthesized by Metabion International AG (Planegg/Steinkirchen, Germany) with annealing temperatures of 60°C. Primers for qPCR were the followings, for *B2-microglobulin*: (*B2m)*-Forward: 5’-ATGGGAAGCCGAACATACTG-3’ and *B2m*-Reverse: 5’-CAGTCTCAGTGGGGGTGAAT-3’ (amplicon 176bp); for *Dll1*: *Dll1*-Forward: 5’-TGGACAGTTGCTTTGAAGAGTA-3’ and *Dll1*-Reverse: 5’-CAAGGAAAGACTGGCTCATAGT-3’ (amplicon 113bp), and for *Notch1*: *Notch1*-Forward: 5’- CCTGACAACTTGAGCCAGTAA-3’ and *Notch1*-Reverse: 5’-CGTCTGTCCTCAGTTGGATTT-3’ (amplicon 118bp). For each of the examined mRNAs, normal and mock reverse transcribed samples (in the absence of reverse transcriptase), as well as no template controls (total mix without cDNA) were run. qPCR was carried out as detailed by Juknat et al., [31]. Expression levels of genes were normalized to the reference gene *B2m* and expressed as fold change using the comparative cycle of threshold method as reported earlier [31]. All qPCR reactions were performed in duplicates and were repeated 3–4 times using different mRNA preparations.

### miRNA qPCR analysis

For miRNA expression studies, cDNA synthesis was performed using the miRCURY LNA Universal cDNA Synthesis kit II (Exiqon, Woburn, MA), according to manufacturer’s instructions. miRNA real time qPCR was carried out using the ExiLENT SYBR Green master mix (Exiqon) and specific primers for mmu-miR-103a-3p (MIMAT0000101; Exiqon # 204063), mmu-miR-34a-5p (MIMAT0000542; Exiqon # 204486), mmu-miR-449a-5p (MIMAT0001542; Exiqon # 204481) and mmu-miR-191-5p (MIMAT0000221; Exiqon # 204306). The qPCR reactions were subjected to an initial activation step at 95°C of 10 min, followed by 40 cycles at 95°C for 10 s and at 60°C for 1 min. For quality control, synthetic RNA spike-in (UniSp6) was added to all RNA samples before reverse transcription and the UniSP6 was used to monitor the efficiency of the RT reaction. Expression of miRNAs was normalized using the means of the expression of miR-103a-3p and miR-191-5p, considered to be the most suitable and stable reference genes.

For the expression studies of miR-146a and miR-155-5p, RNA was reverse-transcribed using miScript II RT Kit (Qiagen), according to the manufacturer’s instructions. Real time qPCR was carried out using miScript SYBR Green PCR Kit (Qiagen), which contains the miScript Universal primer. Primer sequences used were the followings, for miR-146a-5p: 5’-TGAGAACTGAATTCCATGGGT-3’ (MIMAT0000158) and for miR-155-5p: 5’-TTAATGCTAATTGTGATAGGGGT-3’ (MIMAT0000165). miRNA expression was normalized to the reference gene U6 snRNA (U6 small nuclear RNA; NR_003027.2; 5’-GATGACACGCAAATTCGTGAA-3’), which was found to be expressed at high levels and showed no changes under the experimental conditions used in the present study. Real time qPCR reactions were performed at 95°C for 15 min, followed by 40 cycles at 94°C for 15 s, at 55°C for 30 s and at 70°C for 30 s. Melting curve was generated at the end of each run, in order to ensure product specificity. Fold change values in miRNA expression were calculated using the comparative cycle of threshold method as previously described [31]. All qPCR reactions were performed in duplicates in a Rotor-Gene 3000 instrument and were repeated 3–4 times using different miRNA preparations.

### miRNA target predictions

Mature miRNA sequences were obtained from miRBase v21 (http://www.mirbase.org/). The prediction tools MiRTarBase (http://mirtarbase.mbc.nctu.edu.tw) and TarBase v7.0 (http://diana.imis.athena-innovation.gr/DianaTools/index.php?r=tarbase/index) were first chosen as they contain experimental evidence for the predicted miRNA targets. Other algorithms used were TargetScan v7.1 (http://www.targetscan.org/), miRanda, Release 2010 (http://www.microrna.org/; [57]) and miRDB (http://mirdb.org/miRDB/), computational programs based on seed match and conservation [58]. Sequence conservation was verified using UCSC Genome Browser (https://genome.ucsc.edu/). Sequence alignment was examined using the NCBI Basic Local Alignment Search Tool (BLAST) (http://blast.ncbi.nlm.nih.gov/Blast.cgi). Given the high false positive rates for miRNA target prediction, a target was considered as potential if it appeared in at least three of the algorithms used.

### Ingenuity pathway analysis

Pathway and global functional analysis were performed using Ingenuity Pathway Analysis (IPA), an online bioinformatics tool (Ingenuity Systems, QIAGEN Bioinformatics, Redwood City, CA, USA; www.qiagen.com/ingenuity). A data set containing the miRNA and gene identifiers as well as their corresponding expression values, were loaded to the application and each identifier was mapped using the Ingenuity Knowledge Base (IKB). The IKB analyses identify the biological functions as well as the pathways from the IPA library that are most significant to the data set. A cut-off threshold of p ≤0.005 for differential expression was used to build the networks using the IPA tools.

### Statistical analysis

qPCR data is presented throughout the manuscript as fold change and is expressed as the mean ± SEM of 3–4 independent experiments. Statistical significance is analyzed using one way analysis of variance (ANOVA), followed by Tukey post-hoc test. p<0.05 was considered significant. GraphPad Prism 6.0 software (La Jolla, CA, USA) was used for statistical analysis of the data.

## Results

### LPS- and cannabinoid-differentially expressed miRNAs in BV-2 microglial cells

We used a high-throughput sequencing approach (miRNAseq) to analyze the expression of miRNAs in CBD-, THC-, LPS-, CBD+LPS, THC+LPS-treated microglial cells. Samples of miRNA were prepared from BV-2 microglial cells that were pre-incubated for 2 h with 10 μM CBD or 10 μM THC, followed by the addition of LPS (100 ng/ml) to the medium for another 4 h. It is important to note that, according to our previous results, CBD or THC treatment at 10 μM did not significantly affect the viability of BV-2 microglial cells during this 6 h incubation [28].

miRNA sequencing analysis revealed that 31 miRNAs were differentially regulated across the treatments (FDR 0.1; Table 1). Only the miRNAs reaching the criteria of p≤0.005 and fold change of 1.5 were considered for further analysis. They are shown as underlined (for downregulated miRNAs) or with an asterisk (for upregulated miRNAs) in Table 1. As can be seen, there are some miRNAs that although showing a fold change higher than 1.5, did not reach the cut-off threshold of p≤0.005.

**Table 1:** miRNA expression after treatment with LPS and cannabinoids, expressed as fold change versus control.

MicroRNA	Accession^#^	LPS	CBD	CBD+LPS	THC	THC+LPS
mmu-miR-155-3p	MIMAT0016993	28.92*	0.90	24.59*	0.70	21.91*
mmu-miR-155-5p	MIMAT0000165	9.68*	1.35	8.36*	0.64	8.99*
mmu-mir-155	MI0000177	6.51*	1.37	5.26*	0.69	6.43*
mmu-miR-451b	MI0021960	8.40*	11.97*	6.63	7.00	9.24*
mmu-miR-10b-5p	MIMAT0000208	7.40*	1.81	1.38	0.62	5.09*
mmu-miR-146a-3p	MIMAT0016989	5.52*	0.68	3.38*	0.92	3.56*
mmu-miR-21a-3p	MIMAT0004628	3.32*	1.04	2.82*	1.01	2.97*
mmu-mir-21a	MI0000569	2.97*	1.02	2.96*	1.18	3.32*
mmu-miR-21a-5p	MIMAT0000530	2.11*	1.15	1.70*	1.09	1.93*
mmu-miR-221-5p	MIMAT0017060	2.44*	0.69	1.95*	0.97	2.17*
mmu-mir-29c	MI0000577	2.40	1.39	2.53	1.82	3.76*
mmu-mir-20a	MI0000568	2.31*	1.08	1.96	0.84	2.6*
mmu-miR-20a-3p	MIMAT0004627	2.14*	1.22	1.61	0.98	1.82
mmu-mir-222	MI0000710	1.92*	0.83	1.73*	1.02	1.84*
mmu-miR-145a-3p	MIMAT0004534	1.81*	1.17	1.73*	0.99	1.92*
mmu-miR-449a-5p	MIMAT0001542	15.62	36.66*	25.15*	7.96	6.27
mmu-mir-34a	MI0000584	1.46	7.23*	4.48	2.37	2.67
mmu-mir-5112	MI0018021	1.09	2.75*	1.01	1.34	0.96
mmu-mir-181b-1	MI0000723	1.06	3.10*	1.92	1.07	1.21
mmu-miR-5129-5p	MIMAT0020640	1.05	0.03	0.68	1.20	0.51
mmu-mir-696	MI0004677	0.89	0.39	0.44	0.73	0.70
mmu-mir-3096b	MI0016969	0.84	2.02*	1.02	0.84	1.02
mmu-miR-3108-3p	MIMAT0014948	0.78	0.07	2.05	1.79	0.85
mmu-miR-3962	MI0016967	0.78	0.03	0.44	0.75	0.69
mmu-mir-3090	MI0014083	0.71	1.76	0.19	0.62	0.61
mmu-miR-6351	MI0021879	0.69	0.01	0.34	1.08	0.64
mmu-miR-6384	MI0021915	0.43	5.45*	0.82	0.33	0.59
mmu-mir-666	MI0004553	0.43	0.12	0.53	1.21	0.52
mmu-mir-325	MI0000597	0.39	2.72*	0.72	0.97	0.40
mmu-miR-25-5p	MIMAT0017049	0.36	1	0.33	0.61	0.42
mmu-miR-5123	MI0018034	0.07	0.29	0.63	0.17	0.19

^#^Accession number for the corresponding mmu-miRNAs, available at http://mirbase.org/; significantly upregulated miRNAs are marked with an asterisk; significantly downregulated miRNAs are underlined (p≤0.005)

**Fig 1:**
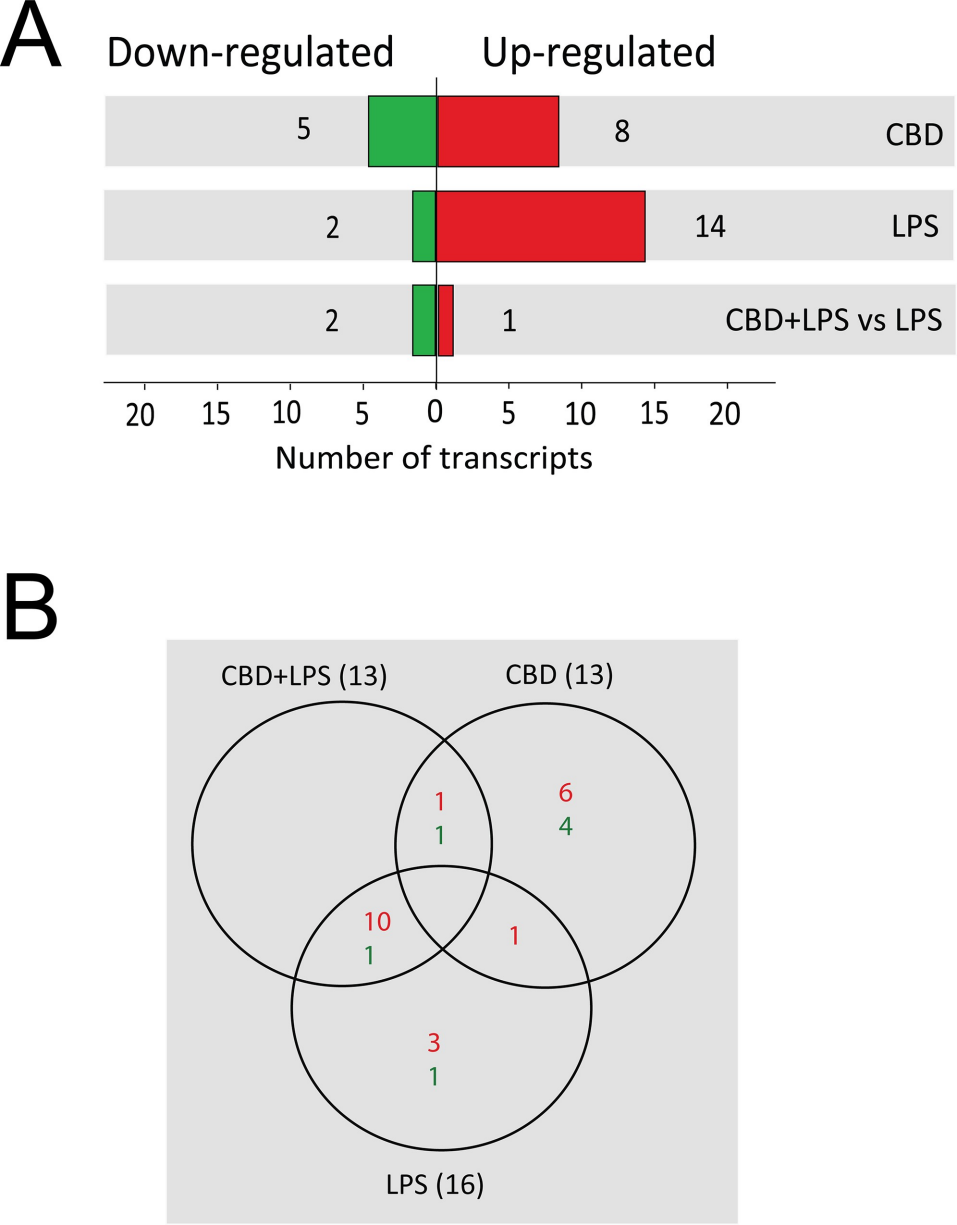
Deep sequencing analysis of the miRNA of BV-2 cells following treatment with LPS and cannabinoids. miRNA was extracted from BV-2 cells treated with LPS and cannabinoids and subjected to deep sequencing analysis as described in Methods. (A) Number of differentially expressed miRNAs that were either significantly upregulated (red) or downregulated (green) across the different treatment conditions versus control untreated cells and versus LPS (p≤0.005). The differentially expressed miRNAs were identified by using a threshold of a 1.5-fold change. (B) Venn diagrams representing the numbers of BV-2 microglial miRNAs that were either upregulated or downregulated at a significance of p≤0.005 following treatments with LPS, CBD and CBD + LPS.

Analyzing the 16 miRNAs affected by LPS stimulation (Fig 1A and 1B; 52% of the total), we found that 14 miRNAs (87.5% of the total LPS-affected miRNAs) were significantly upregulated and that 2 miRNAs (12.5%) were significantly downregulated by the treatment (miR-5123 and miR-25-5p) (p≤0.005; Table 1). Under LPS stimulation, miR-155-3p was the most significantly upregulated miRNA (by 29-fold versus control), followed by others including miR-155-5p (by 9.7-fold), miR-451b (by 8.4-fold), miR-10b-5p (by 7.4-fold), miR-146a-3p (by 5.5-fold) and miR-21a-3p (by 3.3-fold) (p≤0.005; Table 1).

Most of the LPS-upregulated miRNAs were also significantly upregulated by CBD+LPS (10 miRNAs) as shown in Fig 1B, and by THC+LPS (13 miRNAs; Table 1). Moreover, in general, the addition of CBD or THC did not significantly affect the stimulatory effect of LPS. Only one miRNA, miR-10b-5p, that was upregulated by LPS stimulation (by 7.4-fold; p≤0.005; Table 1), was significantly downregulated by CBD + LPS (reduced by 81% when comparing CBD + LPS *vs* LPS; p≤0.005).

Following CBD treatment, the expression of 13 out of the 31 miRNAs (42% of the total) was significantly changed; 8 miRNAs (61.5% of the total CBD-affected miRNAs) were significantly upregulated by more than 2-fold and 5 miRNAs (38.5%) were downregulated by more than 50% compared to the control values (p≤0.005; Fig 1A and 1B). The miRNAs upregulated to the highest extent by CBD treatment were miR-449a-5p (by 37-fold *vs* control), miR-451b (by 12-fold), miR-34a (by 7.2-fold), miR-6384 (by 5.5-fold) and miR-181b-1 (by 3.1-fold) (p≤0.005; Table 1). The downregulated miRNAs included miR-6351 (reduced by 99%), miR-5129-5p (by 97%), miR-3962 (by 97%), miR-666 (by 88%) and miR-696 (by 61%) (p≤0.005; Table 1).

Interestingly, THC treatment only induced changes in one of these miRNAs (miR-5123 downregulated by 83%; p≤0.005; Table 1). From the 5 miRNAs (miR-451b, miR-29c, miR-449a-5p, miR-34a and miR-3108-3p) that show an increase (1.5-fold change) in the expression after THC treatment, only miR-451b could be considered statistically significant if we change the cut-off threshold to p≤0.05. The other miRNAs were found to be not statistically significant.

### Role of CBD in the expression of miR-146a and miR-155-5p in LPS-activated microglial cells

**Fig 2:**
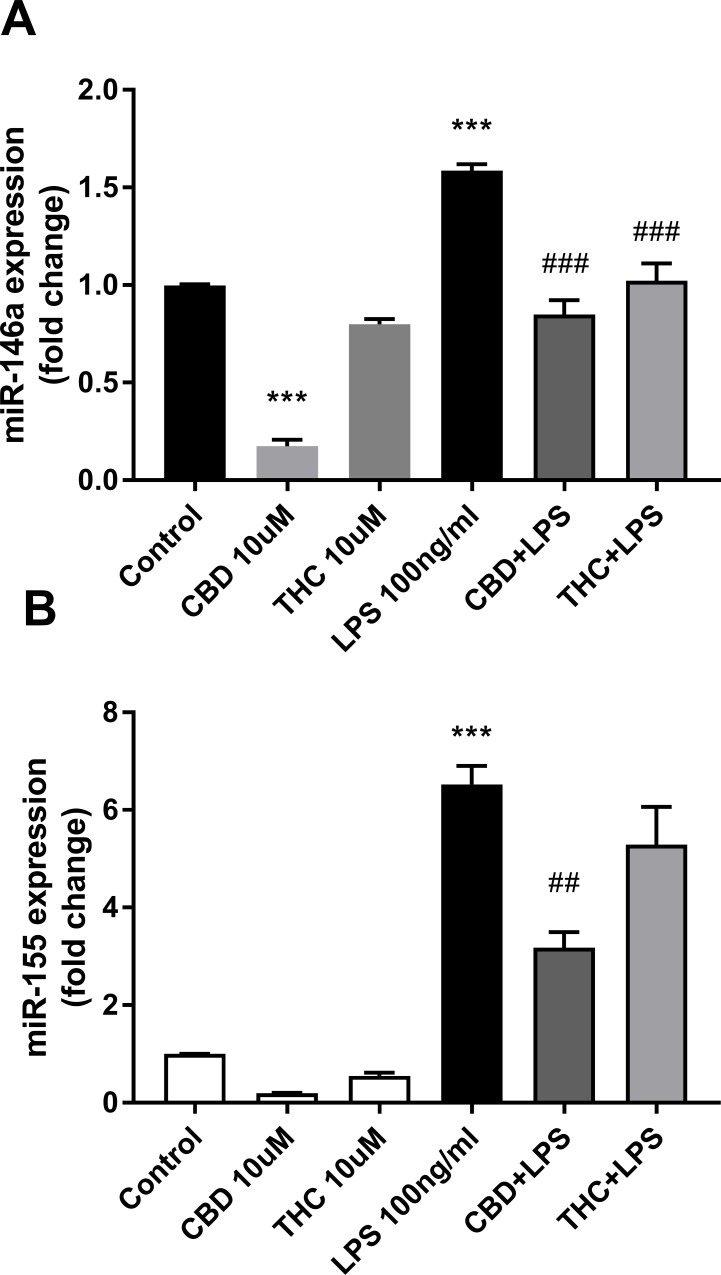
CBD downregulates the expression of miR-146a-5p and miR-155-5p in LPS-activated microglial cells. Cells were treated for 2 h with the indicated concentrations of cannabinoids and then activated for 4 h with 100 ng/ml LPS. Cells were harvested and miRNA was extracted for qPCR analysis. qPCR data were plotted as the mean ± SEM of three to four independent experiments. (A) One-way ANOVA F(5,12) = 78.66 p < 0.001; (B) one-way ANOVA F(5,18) = 49.61 p < 0.001. Tukey multiple comparison post hoc test: ***p < 0.001 vs. control; ##p < 0.01 and ###p < 0.001 vs. LPS.

Table 1 shows that several miRNAs are upregulated by LPS treatment. This included miR-155, miR-146a and miR-21. This result is in agreement with the finding that this trio of miRNAs is involved in many immune and inflammatory pathways, known to control TLR signaling [4, 10–11]. The effects of cannabinoids on the level of expression of miR-146a and miR-155-5p were evaluated by qPCR. The modulation of the expression of both miRNAs show a more pronounced effect of 10 μM CBD as compared to 10 μM THC (Fig 2).

In resting cells, miRNA 146a expression was highly and significantly downregulated by CBD (by 83%; p<0.001; Fig 2A). In agreement with the sequencing data, both miR-146a and miR-155-5p were upregulated by treatment with LPS (by 1.6-fold; p<0.001, and 6.5-fold; p<0.001, respectively). This upregulation is reduced by pretreatment with CBD (by 47%; p<0.001, and by 51%; p<0.01, respectively) (Fig 2A and 2B). At the concentration tested, the effect of THC was lower than that of CBD; the LPS-upregulated miR-146a was reduced by 37% (THC + LPS; p<0.001; Fig 2A). THC pretreatment did not show any significant effect on LPS-upregulated miR-155-5p (THC + LPS; Fig 2B).

Target prediction was performed for both, miR-146a and miR-155. S1 and S2 Tables contain a list of genes, validated according to miRTarBase and TarBase prediction tools. These targets were chosen using bioinformatics tools as described in Methods. Indeed, we found that many of these genes are significantly affected by LPS as well as by the presence of cannabinoids during LPS activation, according to the RNAseq data (fold change set on ≥1.4-fold or ≥40% reduction; p≤0.005). Selected target genes were found to be involved in TLR signaling (*Irak2*, *Irak3*, *Ikbk*ε*/Ikkε*, *Cot/Tpl2/Map3k8*, *Inpp5d/Ship1*), cytokine-mediated signaling (*Il6*, *Il6ra*), regulation of transcription (*Stat1*, *Pparg*, *Tp53inp1/Trp53inp1*), NF-κB signaling (*Relb*, *Ikbkε/Ikkε*), stress response (*Hmox1*, *Tp53inp1/Trp53inp1*, *Nrf2/Nfe2l2*), signal transduction (*Cish*, *Socs1*), Notch pathway (*Notch1*, *Notch2*, *Tspan14*), lipid signaling (*Lipin1*, *S1pr1*) as well as cell death and apoptosis (*Fas*, *Cflar*).

S1 Table shows that the cannabinoids reduced the expression of LPS-upregulated *Il6* mRNA as previously reported by us and others [28]. Among other targets, we were interested in *Pparg*, even though it was identified as a prediction target for miR-146a by only one prediction tool, the miRTarBase. *Pparg* mRNA expression was observed to be inversely correlated with miR-146a expression in LPS-stimulated microglia, following the canonical repression pattern (S1 Table). Indeed, we previously showed that LPS downregulates the mRNA expression of both *Pparg1* and *Pparg2* isoforms, in BV-2 microglial cells [31], in agreement with reports showing that NF-κB drives down Pparg expression in LPS-stimulated macrophages [59]. However, CBD did not exhibit any significant effect on *Pparg* mRNA expression in BV-2 microglial cells eventhough Pparg has been identified as a molecular target for CBD [60].

### miR-34a/449a is upregulated in microglial cells in response to CBD treatment

One of the miRNAs that attracted our attention was miR-449, that was greatly upregulated by CBD (Table 1). The miR-449 cluster, containing the highly conserved miR-449a, miR-449b and miR-449c, was found to share strong sequence homologies with the miR-34 family (miR-34a, miR-34b and miR-34c) and therefore, classified as the miR-34/449 family. MiR-34a and miR-449a share the same seed sequence (GGCAGUG), suggesting similar mRNA targets [61]. However, despite these similarities, miR-449 and miR-34 seem to have distinct functions and to be regulated differentially [62]. MiR-34a is ubiquitously expressed, except in lung tissue, showing the highest levels in brain tissues, while miR-34b/c and miR-449a/b/c are mainly expressed in lung and testis, demonstrating tissue-specific functions [61, 63].

**Fig 3:**
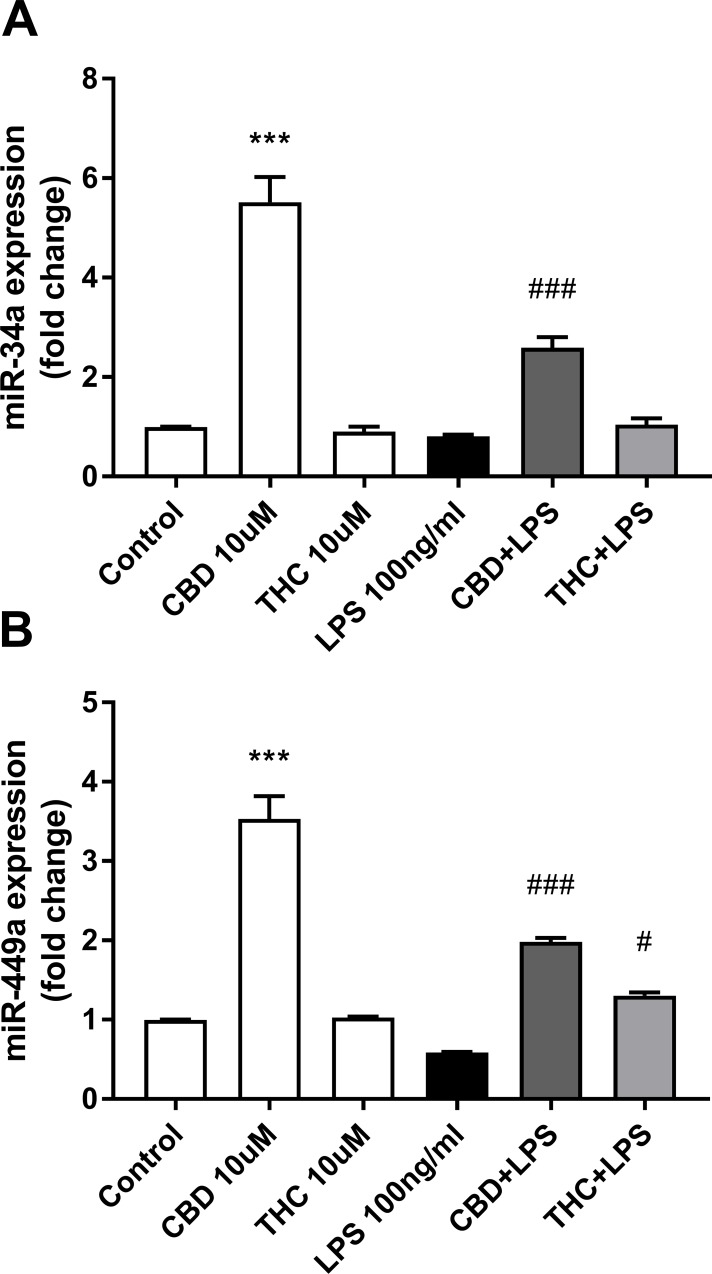
CBD upregulates the expression of miR-34a/449a in resting microglial cells. Conditions are as indicated in Fig 2. qPCR data were plotted as the mean ± SEM of three to four independent experiments. (A) One-way ANOVA F(5,19) = 62.78 p < 0.001; (B) one-way ANOVA F(5,12) = 78.31 p < 0.001. Tukey multiple comparison post hoc test: ***p < 0.001 vs. control; #p < 0.05 and ###p < 0.001 vs. LPS.

Analysis of miR-34a and miR-449a expressions by qPCR in BV-2 microglial cells showed similar profiles (Fig 3).

**Fig 4:**
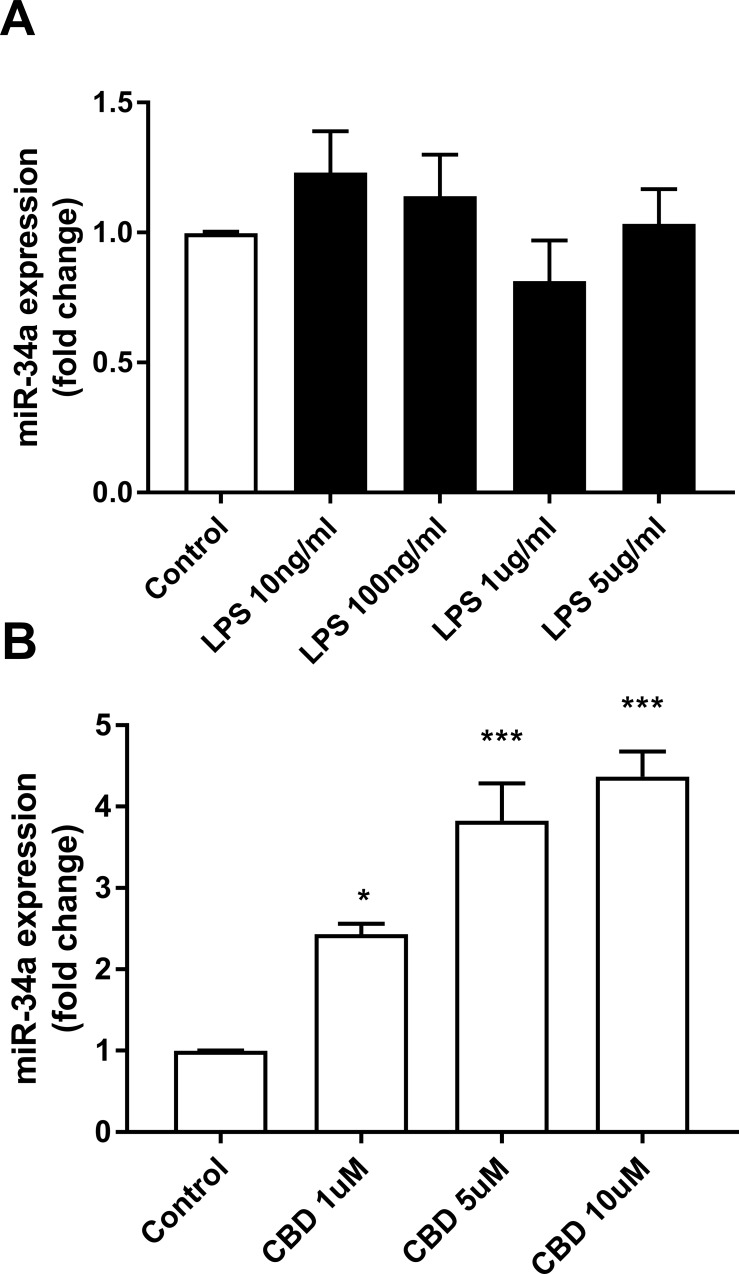
miR-34a expression is not affected by LPS, but upregulated by CBD. Cells were treated for 4 h with the indicated concentrations of LPS or for 6 h with the indicated concentrations of CBD. Cells were harvested and miRNA extracted for qPCR analysis. qPCR data were plotted as the mean ± SEM of three to four independent experiments. (A) One-way ANOVA F(4,10) = 1.339 not significant; (B) one-way ANOVA F(3,8) = 28.12 p < 0.001. Tukey multiple comparison post hoc test: *p < 0.05 and ***p < 0.001 vs. control.

Both, miR-34a and miR-449a were upregulated by CBD (by 5.5-fold; p<0.001; Fig 3A and by 3.5-fold; p<0.001; Fig 3B respectively), although miR-449a to a lower degree when compared to the sequencing data (37-fold; p≤0.005; Table 1). As pointed out for miR-146a and miR-155-5p, we observed also for miR-34a and miR-449a a more pronounced effect of CBD as compared with THC. Using qPCR, we examined miR-34a expression in BV-2 cells stimulated with different doses of LPS (0 to 5 μg/ml) and found out that it was not affected by LPS at any dose (Fig 4A); on the other hand, it was upregulated by CBD treatment in a dose dependent manner (Fig 4B). It is interesting to point out that THC does not affect the expression of miR-34a at all the concentrations tested (0 to 10 μM) under our experimental conditions (data not shown).

Potential target genes for miR-34a-5p were chosen using the target prediction tools described in Methods. S3 Table shows a selection of miR-34a target genes which were found to be affected by cannabinoids, LPS, or their combinations. Most of these target genes encode factors required for cell cycle regulation (*Ccnd1*, *Ccne2*, *Cdkn1a/p21*, *E2f5*, *Cdc25a*), Notch signaling (*Notch1*, *Notch2*, *Dll1*), inflammation (*Il6*, *Tnf*), Wnt/β-catenin signaling (*Wnt1*), stress response (*Slc7a11/xCT*, *Ddit4*, *Ndrg1*, *Tp53inp1/Trp53inp1*), membrane transport (*Aqp9*, *Slc7a11/xCT*), apoptosis (*Gas1*, *Mdm2*, *Pdgfrb*), regulation of transcription (*Rora*, *Rela*, *Foxp1*, *Hes2*, *Tp53inp1/Trp53inp1*), and senescence (*Hbp1*). Among these targets, the expression of the cell cycle-related transcripts (*Ccnd1*, *Ccne2*, *E2f5*, *Cdc25a*) was found to be inversely correlated with miR-34a expression in CBD-treated cells (S3 Table).

**Fig 5:**
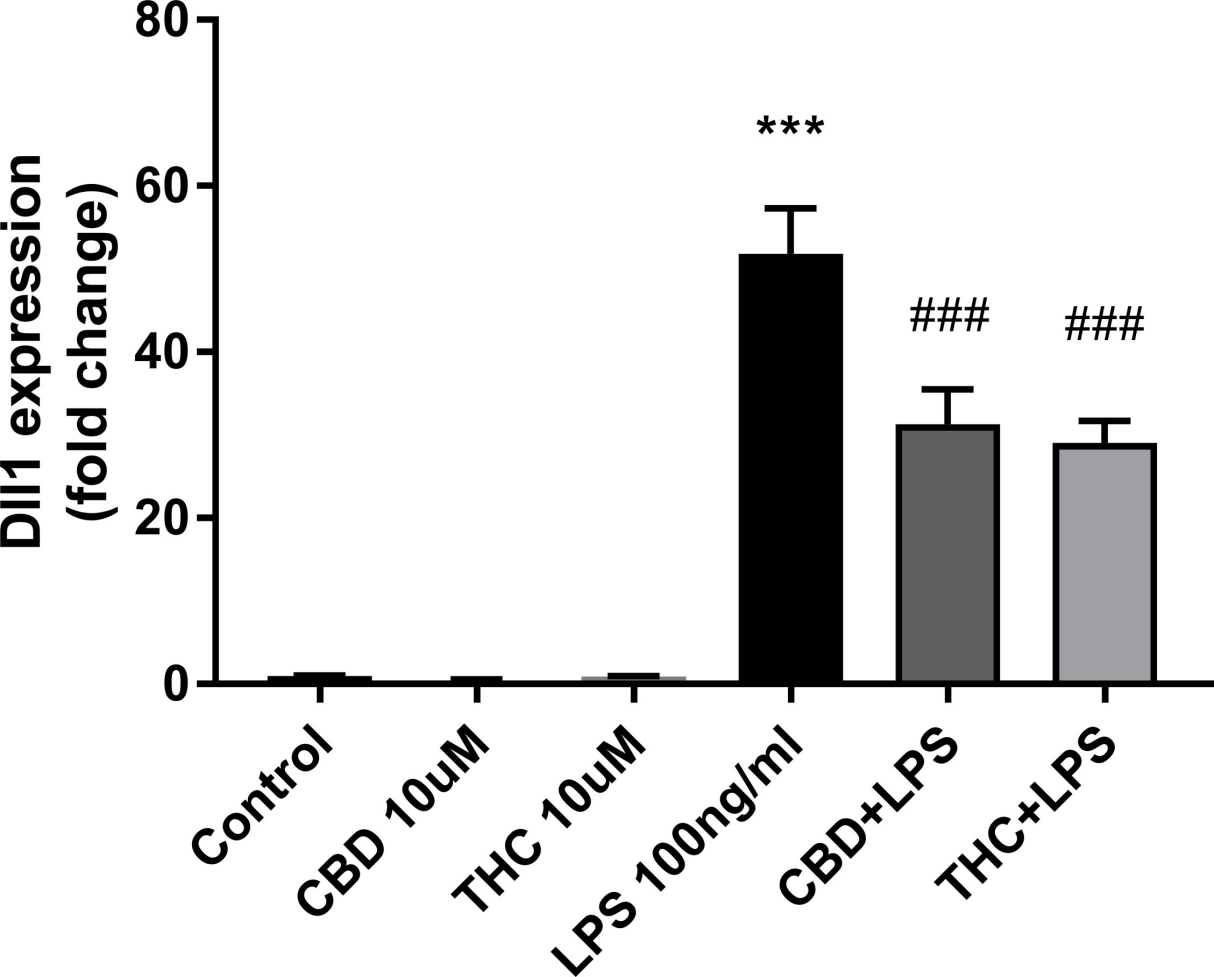
CBD and THC downregulate the expression of the Notch ligand Dll1 in LPS-activated microglial cells. Conditions are as indicated in Fig 2. qPCR data were plotted as the mean ± SEM of three to four independent experiments. One-way ANOVA F(5,24) = 50.54 p < 0.001. Tukey multiple comparison post hoc test: ***p < 0.001 vs. control; ###p < 0.001 vs. LPS.

Among the miR-34a target genes, we validated the Notch ligand *Dll1* by qPCR (Fig 5).

Surprisingly, in resting cells, *Dll1* expression was downregulated by CBD by 56%, a result that was found to be not statistically significant, in contrast to the upregulation pattern observed in the sequencing data (upregulation by 11-fold; p≤ 0.0005; S3 Table). Analyzing the effect of LPS, we observed an upregulation of *Dll1* expression by LPS (by 52-fold; p<0.001), which was decreased by pretreatment with CBD (by 40%; p<0.001) and with THC (by 44%; p<0.001) (Fig 5).

### Network analysis and signaling pathways

**Fig 6:**
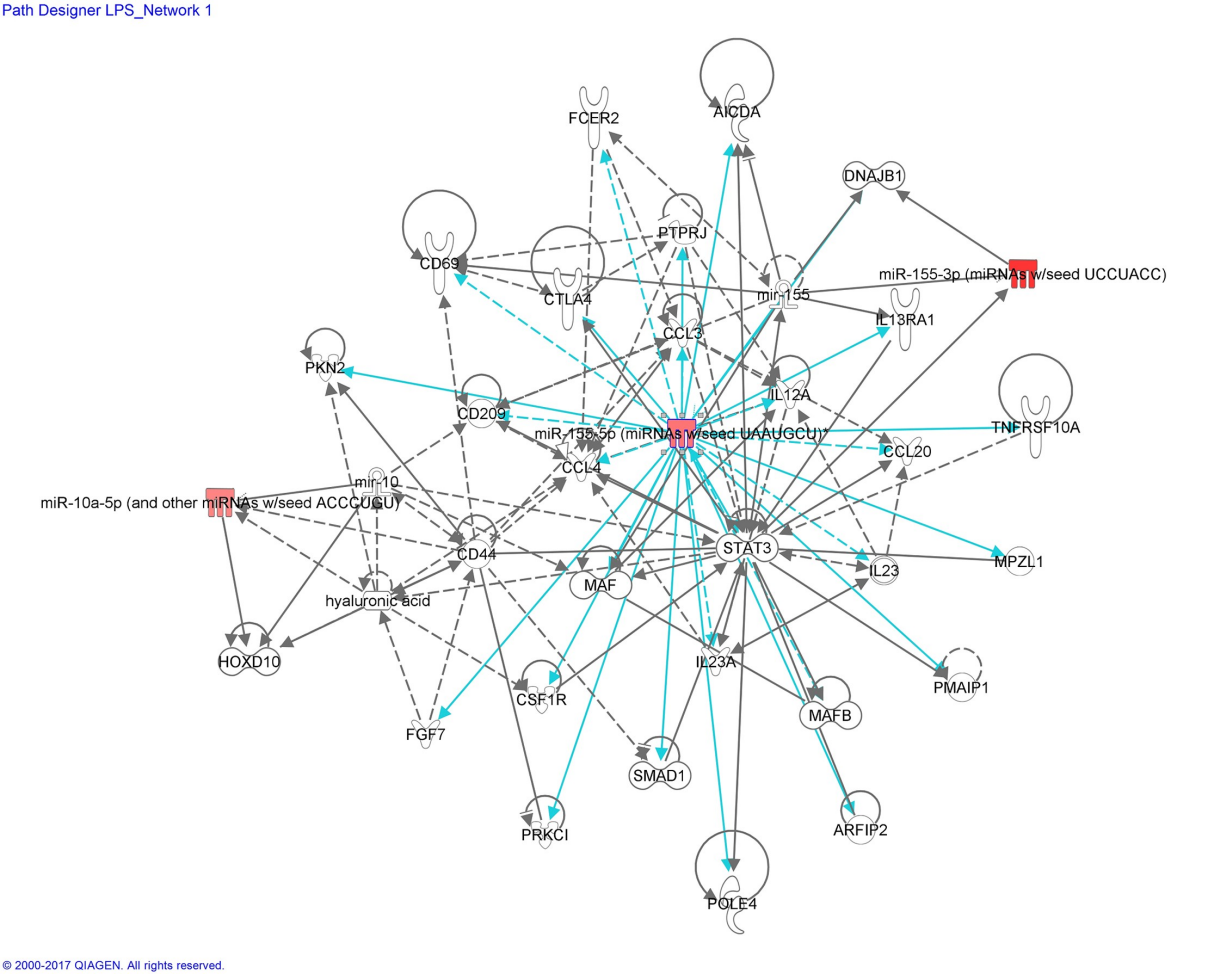
miRNA:mRNA gene network associated to LPS-stimulated microglial cells, identified by Ingenuity Pathway Analysis (IPA)—Network LPS_miR-155-5p. Network analysis of the genes whose expression was affected by LPS was performed using the Ingenuity software. Each network displays the genes/gene products as nodes (different shapes representing the functional classes of gene products) and the biological relationships between the nodes as lines. Full lines represent direct interactions and broken lines, indirect interactions. Lines in gray represent interactions shown in the literature. Lines in turquoise represent the interactions provided by our mRNA/miRNA sequencing data. The color intensity of the mature miRNA node indicates the degree of upregulation (red) of the respective miRNA.

**Fig 7:**
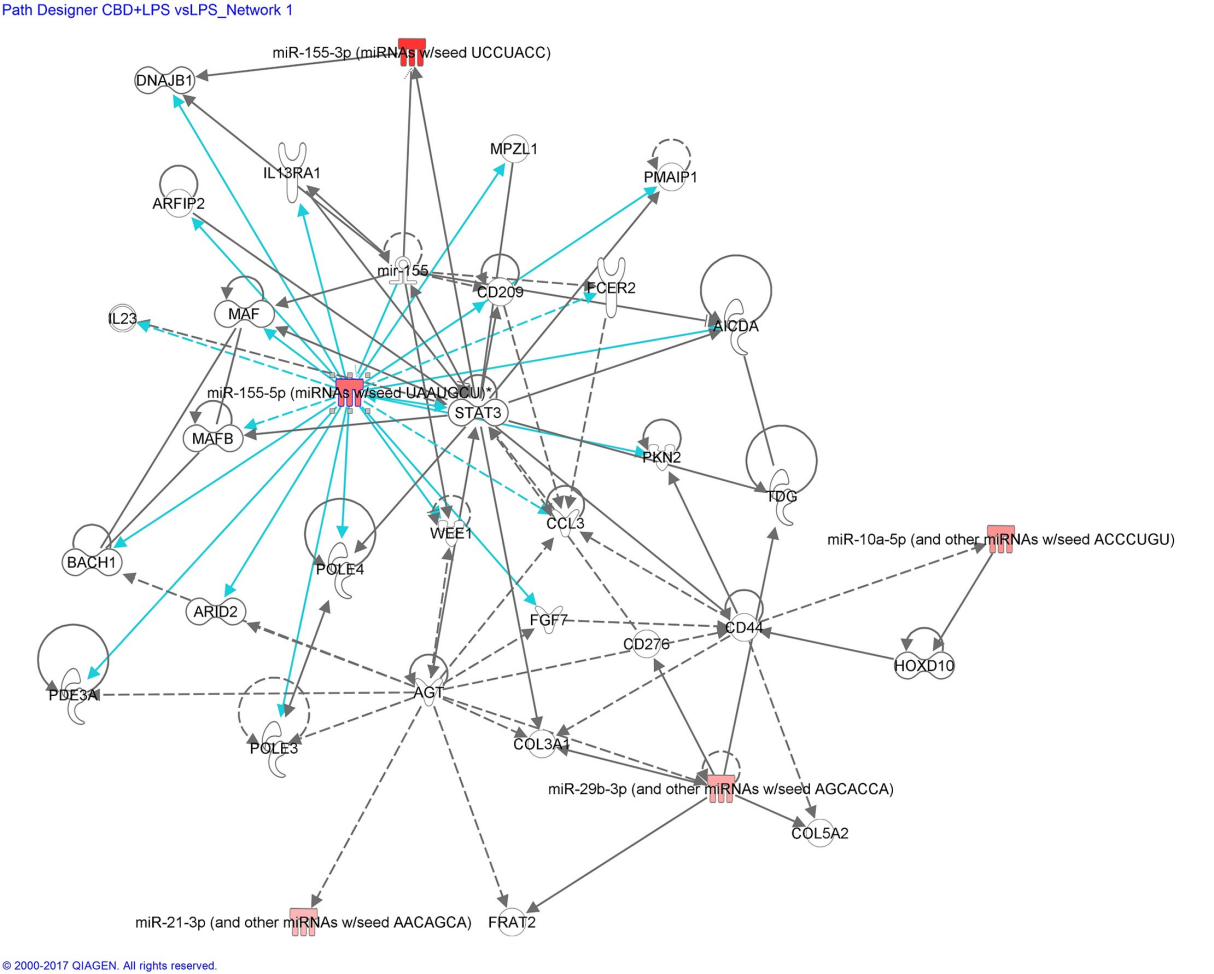
miRNA:mRNA gene network showing the effect of CBD on LPS-stimulated microglial cells, identified by IPA—Network CBD + LPS vs LPS_miR-155-5p. Details are as indicated in Fig 6.

**Fig 8:**
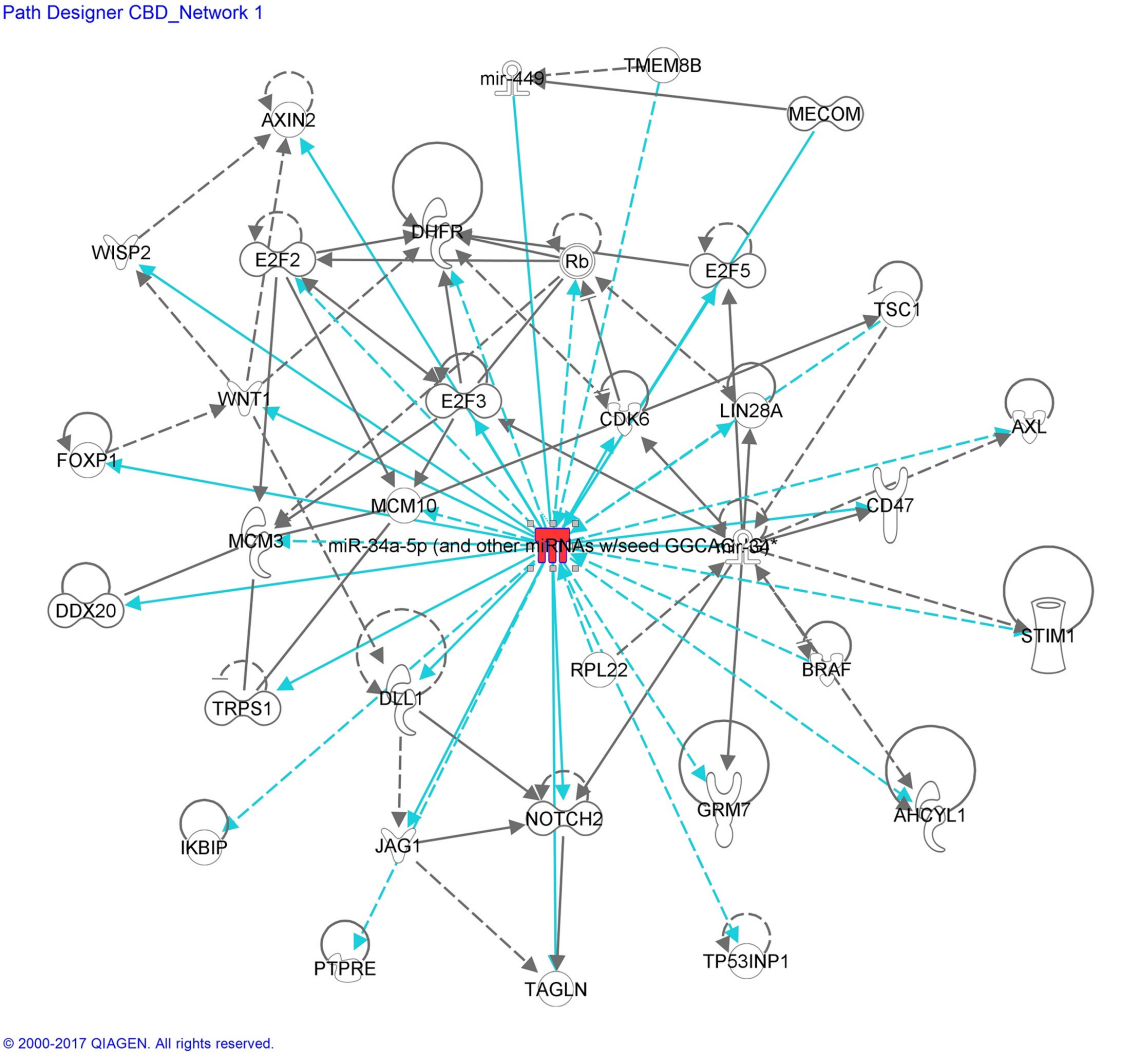
miRNA:mRNA gene network associated to CBD-treated microglial cells, identified by IPA—Network CBD_miR-34a-5p. Details are as indicated in Fig 6.

The differential miRNA expression patterns, revealed by miRNAseq of BV-2 microglial cells treated with LPS and/or cannabinoids, were analyzed using IPA, which integrates published findings on biologically meaningful genetic or molecular gene/gene product interactions and identifies new targets as well as functionally related gene networks. Differential miRNA expression values (*e*.*g*.; LPS *vs* control, CBD *vs* control or CBD + LPS *vs* LPS) were entered into IPA to determine the most highly regulated networks of miRNA-mRNA interactions and to highlight the biological processes that are relevant to each of the treatments. Genes that met the p value cutoff of 0.005 for differential expression were used to build the gene networks using IPA tools. Networks describing the relationships between miRNAs and a subset of genes and their related transcripts are presented in Figs 6–8.

Interactome analysis of LPS and associated miRNA:mRNA gene network is shown in Fig 6.

This network has in its central position the mature miRNA-155-5p (and other miRNAs with seed sequence UAAUGCU, including miR-155, considered by IPA as a duplicate). These miRNAs are upregulated by LPS (Table 1; Fig 2B). The LPS-Network interconnects miR-155-5p (possessing 15 edges, *via* direct connections and 9 edges, *via* indirect ones) with cytokines (*Il12a/b*, *Il23a*, *Il13ra1*), chemokines (C-C motif) (*Ccl4*, *Ccl3*, *Ccl20*), cell surface antigen molecules (*Cd209*, *Cd44*, *Cd69*, *Cd23/Fcer2a*, *Cd152/Ctla4*), transcription factors (*Stat3*, *Smad 1*, *MafB*), regulators of apoptotic processes (*Pmaip1/Noxa*), kinases (*Pkn2*, *Prkci*), phosphatases (*Ptprj*), other enzymes (*Aicda/Arp2/Aid*), regulators of signal transduction (*Mpzl1*) and regulators of cytoskeleton organization (*Arfip2/Por1*).

Fig 7 shows miRNA:mRNA interactions for CBD + LPS *vs* LPS.

Mature miR-155-5p appears to play a central role, showing 17 edges of direct connections. However, only three transcripts were exclusively found to be interconnected to miR-155-5p after CBD+LPS treatment: *Bach1*, a transcription factor involved in oxidative stress; *Wee1*, a cell cycle regulator; and *Pole3/Ybl1*, an enzyme with polymerase activity. The other transcripts interconnected to miR-155 after LPS treatment, were already exhibited in Fig 6.

Interactome analysis of CBD and associated miRNA:mRNA gene network is shown in Fig 8.

MiR-34a-5p (and other miRNAs with seed sequence GGCAGUG, including miR-449a-5p considered by IPA as a duplicate of miR-34a-5p) is upregulated by CBD (Table 1; Fig 3A and 3B) and plays a central role in this network, possessing 14 and 13 edges *via* direct and indirect connections. A series of canonical pathways, including those involved in Rb (Retinoblastoma)/E2F cell cycle (*Rb*, *E2f2*, *E2f3*, *E2f5*, *Cdk6*, *DHFR*), Notch (*Dll1*, *Notch 2*, *Jag1*), Wnt (*Wnt*, *Axin2*, *Wisp2/Ccn5*) and NF-κB (*Ikbip*) were found to participate in this network. Other affected genes are related to apoptosis (*Ddx20*, *Ikbip*), integrin-associated signal transduccion (*Cd47*), stress protein with antioxidant-associated tumor suppressive function (*Tp53inp1/Trp53inp1*), calcium signaling (*Stim1*), as well as those related to proliferation and survival (*Lin28a*).

## Discussion

In the present study, we identified a repertoire of miRNAs that are differentially modulated by cannabinoids and/or LPS treatments in microglial BV-2 cells.

It attracted our attention the poor effect exhibited by THC in our experimental model. Only miR-5123 is significantly affected by THC. In addition, 14 miRNAs were found to be upregulated by THC+LPS treatment, from them 13 miRNAs were also affected by LPS more or less, to the same extent. THC reduces the expression of LPS-upregulated miR-146a but not of LPS-upregulated miR-155-5p. THC+LPS treatment increased the expression of miR-449a-5p but not of miR-34a-5p. Comparing the cannabinoids tested, 10 μM CBD appears to have a much greater effect than 10 μM THC on the expression of these miRNAs. These results are in line with the more robust effects of CBD on mRNA regulation in resting/surveillant and LPS-activated microglial cells, as compared with THC, shown by us previously [28,31,33].

Here we show that CBD strongly downregulates miR-146a in resting cells and that both CBD and THC inhibit the LPS-upregulated expression of this miRNA. Once LPS induces NF-κB signaling through the MyD88-dependent pathway, activated NF-κB induces the transcription of many genes, including miR-146a and the major pro-inflammatory cytokines *Il-6* and *Il-1*β.

In addition, an excessive activation of NF-κB induces miR-146a to act as a negative regulator of inflammation, leading to a reduction of NF-κB transcriptional activity through targeting Irak1 and Traf6, and through suppression of NF-κB inducible genes such as *Il-6* and *Il-1*β [64]. It is important to note that the key role of miR-146a as a negative feed-back regulator is fundamental to prevent a disregulated inflammation process and to control its magnitude, but miR-146a is also upregulated during immune activation through the increase in NF-κB activity [65]. We suggest that CBD is downregulating miR-146a expression by targeting NF-κB signaling, and by this way, decreasing the mRNA levels of LPS-upregulated *Il-6* and *Il-1*β in BV-2 microglial cells [28]. In this regard, our group previously demonstrated that CBD reduces the activity of the NF-κB signaling pathway, by partially decreasing LPS-induced degradation of IκB as well as by reducing LPS-induced NF-κB p65 subunit phosphorylation [28]. In addition, CBD decreased the level of Il-6 and Il-1β proteins, providing evidence of the anti-inflammatory effects of CBD as mediated *via* NF-κB [28]. Although Irak1 and Traf6 were identified as direct targets for miR-146a, we did not find these adapter molecules through our target prediction tools. Moreover, studies from our group showed that 10 μM CBD did not affect the level of Irak1 and IkB proteins as well as the level of phosphorylated p65, in the absence of LPS, in 6h experiments [28]. However, we demonstrated that CBD partially decreased LPS-induced degradation of Irak1 [28], showing a possible participation of miR-146a as a negative regulator. Therefore, more studies are required in order to understand the sequence of events CBD+LPS/miR-146a/Irak1 and the final effect of CBD on NF-κB signaling.

Although miR-146a and miR-155 are co-induced by LPS, their final effects are different. MiR-146a exerts control of the inflammatory response leading to reduced inflammation by fine-tuning TLR signaling rather than abolishing the signal, whereas miR-155 is a pro-inflammatory miRNA, essential in driving the inflammatory phenotype M1 [66]. MiR-155 is the most highly upregulated miRNA in response to inflammatory stimuli, such as LPS and/or IFN-γ, in diverse immune cells [13, 67–68], including microglia [14–15, 69]. Reports show that the main target of miR-155 is the inhibitory protein of inflammation Inpp5d/Ship1 (inositol polyphosphate-5-phosphatase D/Src homology-2 domain-containing inositol 5-phosphatase 1), a negative regulator of TLR4 signaling. Our present results demonstrate that after LPS activation, miR-155-5p expression is upregulated, in a good correlation with the reduction of Inpp5d/Ship1 expression (downregulated by LPS by 60%), leading to inflammation. CBD pretreatment reverses this effect, in a good agreement with its inhibition of the LPS-upregulated miR-155-5p expression. CBD reduces the LPS-increased miR-155-5p expression by 51%, leading to a decrease in Inpp5d/Ship1 degradation (Inpp5d/Ship1 expression nearly reaches control values; p≤0.01). Although Inpp5d/Ship1 is a negative regulator of TLR4, its level of expression after CBD+LPS treatment for 6 h does not arrive to the threshold required to mounting an inflammatory response.

Another target of mir-155 is Ikbkε/Ikkε (inhibitor of kappaB kinase epsilon) which functions in the TLR4/TRIF/MyD88-independent pathway, inducing Ifnb production. Our group showed that CBD decreases the mRNA levels of LPS-upregulated *Ifnb* as well as the LPS-induced release of Ifnb in BV-2 cells, suggesting that CBD exerts its inhibitory activity upstream of Ifnb production [28]. As CBD reverses the LPS-upregulated Ikbkε expression reaching control values and decreases upregulated Ifnb1 mRNA expression (by 70%, p≤0.005; [33]), CBD might be acting at the level of the paralog kinases Ikbkε/Tbk1 (TANK-binding kinase 1), that are activating Irf3 and hence, Ifnb synthesis [70]. Altogether, these results provide evidence for the anti-inflammatory effects of CBD.

Among the miR-155 target transcripts, we find the stress response genes Hmox1 and Bach1, both of them upregulated by CBD (by 3.0-fold; p≤0.01 and by 40%; p≤0.005, respectively) and by CBD+LPS (by 9.4-fold; p≤0.0001 and by 80%; p≤0.0001, respectively). Interestingly, the highly pro-inflammatory miR-155 has been reported to be cytoprotective during inflammation by enhancing the induction of Hmox1 expression *via* inhibition of Bach1 translation in endothelial cells [71]. Moreover, miR-155 has been found to attenuate inflammation and oxidative injury *via* enhancement of Nrf2-mediated *Hmox1* and *Slc7a11/xCT* gene expressions [72]. In addition, our group reported that many transcripts induced by CBD, including *Hmox1* and *Slc7a11/xCT* are classified as Nrf2-mediated oxidative stress response genes [31,33]. This specific Nrf2-mediated redox-regulated gene expression pattern defines the microglial phenotype Mox [18], and suggests that BV-2 cells switch from the inflammatory phenotype M1 in LPS-activated microglia to the Nrf2-mediated Mox in CBD-treated cells.

CBD was found to strongly upregulate miR-34a in resting and in LPS-treated cells. MiR-34a has multiple roles including regulation of cell cycle, apoptosis, differentiation and migration, and its expression is controlled by p53 [73–74]. Accordingly, miR-34a has many potential target genes, among them, the Notch receptors and their ligands, associated with innate immunity and inflammation through Notch-TLR crosstalk [75]. LPS upregulates Notch1 and Notch2 mRNA expressions. In addition, we show that LPS upregulates Notch ligand Dll1 mRNA expression, in line with the findings that activation of microglia or macrophages with TLR4 ligands leads to induction of Notch receptors and their ligands, including Dll1, Dll4 and Jagged1 [75–78]. Our results demonstrate that LPS modulates Notch signaling by inducing the expression of Dll1. In addition, CBD and THC reduce the expression of LPS-upregulated Dll1 showing an effect of cannabinoids on Notch signaling. As CBD+LPS upregulates miR-34a expression and reduces its target gene Dll1, we suggest that miR-34a might be involved in the modulation of Notch signaling pathway, leading to reduced inflammation.

Interestingly, a cross-talk between Notch and Nrf2 signaling has been reported [79], and miR-34a has been demonstrated to be involved in the modulation of the Nrf2 anti-oxidant pathway [72]. In this regard, cellular redox homeostasis in BV-2 microglial cells, might be regulated by redox sensitive miRNAs, including miR-155 and miR-34a, through their modulation of Nrf2-driven gene expression, upregulated by CBD [31,33].

Fig 8 shows other miR-34a potential target genes, specially a group involved in the RB/E2F cell cycle. We found in our RNAseq data, several target genes related to cell cycle regulation, including *Ccnd1*, *Ccne2*, *Cdk6*, *Cdk1a/p21*, *E2F5* and *Cdc25a*, previously shown by us, to be affected by CBD and/or LPS and their combination [31,33]. *Ccnd1*, *Ccne2*, *Cdk1a/p21*, *E2f5* and *Cdk6* are involved in the G_1_/S transition *via* the Rb-E2F signaling pathway, and *Cdc25a* plays a role in assisting both G_1_/S and G_2_/M progressions. We show here that CBD downregulates the mRNA expression of *Ccnd1* (50%, p<0.0001), *Ccne2* (50%, p<0.0001) and *E2f5* (50%, p<0.001), as well as of the dual phosphatase *Cdc25a* (40%, p<0.0001). All together, CBD could induce a cell cycle arrest in the G_1_ phase leading to inhibition of the E2F pathway [80–81]. In addition, CBD upregulates *Cdkn1a/p21* (3.3-fold, p<0.001), that inhibits the activity of cyclinD-Cdk4, preventing Rb phosphorylation during G_1_ to S phase transition. These results confirm that CBD plays a role in cell cycle arrest at the level of the G_1_/S phase transition. Moreover, it has been reported that regulation of the G_1_/S transition through modulation of *Ccnd1* expression represents a central mechanism through which NF-κB/IKK signaling regulates cell growth and proliferation [82]. Our data thus suggests that CBD inhibits proliferation through G1/S phase arrest (involving *Ccnd1* downregulation and *Cdkn1a/p21* upregulation), and that this CBD-inhibitory effect possibly depends on the NF-κB signaling transduction pathway.

It is important to note here that many of the identified miRNA:mRNA interactions do not obey the canonical repression rule, as pointed out by others [14, 68]. For example, *Irak1/2* (a target of miR-146) as well as *Socs1*, *Cebpb* and *IkbkƐ* (targets of miR-155) are upregulated instead of repressed, as would be expected. In this regard, it is accepted that each miRNA has many targets and that each mRNA can be regulated by more than one miRNA, suggesting a more complex regulatory mechanism than an ordinary direct repression of a target gene [83].

In conclusion, the present miRNA expression profiling highlights the role of several miRNAs in modulating inflammatory networks in LPS-activated microglia. Here we show that CBD anti-inflammatory effects are mediated via reduction in the expression of some LPS-activated miRNAs, by targeting NF-κB and IRF3 pathways, as well as by reducing LPS-upregulated *Dll1* expression, involved in Notch-TLR crosstalk signaling. CBD and CBD+LPS effects are found to be linked to targets modulated by miR-146a, miR-155 and miR-34a, especially genes related to inflammation (*Il6* and *Ifnb1*), Notch signaling (*Dll1*), cell cycle regulation (*Ccnd1* and *Cdkn1a/p21*), and Nrf2-mediated oxidative stress response (*Hmox* and *Bach1*). All together, miRNAs affected by CBD and less so by THC, show to be linked to inflammatory pathways, cell cycle arrest and Nrf2-mediated cellular stress.

## Supporting information

S1 Table Selected miR-146a target genes. (PDF)

S2 Table Selected miR-155 target genes. (PDF)

S3 Table Selected miR-34a target genes. (PDF)
