# Supplementary material for: miRNA expression profiles and molecular networks in resting and LPS-activated BV-2 microglia—Effect of cannabinoids
Source: PLoS One. 2019 Feb 11;14(2):e0212039. doi: 10.1371/journal.pone.0212039 (PMC6370221; doi:10.1371/journal.pone.0212039)
Supplement: S1 Table — (PDF) [file pone.0212039.s001.pdf]

S1 Table. Selected miR-146a target genes

Gene Name	Description	Accession*	Fold change (versus control)			Fold change (versus LPS)	
			**LPS**	**CBD**	**THC**	**CBD + LPS**	**THC + LPS**
Immune response and inflammation							
Il6	interleukin 6	MGI:96559	159	3.1	0.3	0.5	0.5
Host defense							
Rsad2	radical S-adenosyl methionine domain containing 2	MGI:1929628	36.6	4.5	0.5	1.4	0.7
TLR signaling							
Irak2	interleukin-1 receptor-associated kinase 2	MGI:2429603	4.0	1.1	1.1	0.9	0.9
Cot/ Tpl2 / Map3k8	mitogen-activated protein kinase kinase kinase 8	MGI:1346878	4.6	1.3	4.0	0.9	0.8
Chemokines							
Cxcr4	chemokine (C-X-C motif) receptor 4	MGI:109563	0.1	4.6	0.2	2.5	4.0
Metabolic							
Nos2	nitric oxide synthase 2, inducible	MGI:97361	553	2.7	0.7	0.9	0.7
Cell death and apoptosis							
Fas	Fas (TNF receptor superfamily member 6)	MGI:95484	22.6	1.8	0.7	0.8	0.8

Notch signaling							
Notch1	notch 1	MGI:97363	6.6	1.3	1.1	1.0	1.1
Regulation of transcription							
Relb	avian reticuloendotheliosis viral (v-rel) oncogene related B	MGI:103289	3.5	1.5	0.9	1.2	0.9
Stat1	signal transducer and activator of transcription 1	MGI:103063	3.1	1.8	0.8	0.8	0.8
Pparg	peroxisome proliferator activated receptor gamma	MGI:97747	0.4	1.2	0.9	0.7	1.0
Stress response							
Vegfa	vascular endothelial growth factor A	MGI:103178	0.8	2.1	1.3	3.2	1.3

* Accession number available at Mouse Genome Informatics (MGI): http://www.informatics.jax.org/
