# Supplementary material for: miRNA expression profiles and molecular networks in resting and LPS-activated BV-2 microglia—Effect of cannabinoids
Source: PLoS One. 2019 Feb 11;14(2):e0212039. doi: 10.1371/journal.pone.0212039 (PMC6370221; doi:10.1371/journal.pone.0212039)
Supplement: S2 Table — (PDF) [file pone.0212039.s002.pdf]

S2 Table. Selected miR-155 target genes

Gene Name	Description	Accession*	Fold change (versus control)			Fold change (versus LPS)	
			LPS	CBD	THC	CBD + LPS	THC + LPS
Immune response and inflammation							
Il6ra	interleukin 6 receptor, alpha	MGI:105304	0.3	0.7	0.9	1.0	1.3
Mr1	major histocompatibility complex, class I-related	MGI:1195463	0.5	1.9	0.6	0.6	1.0
TLR signaling							
Irak3	interleukin-1 receptor-associated kinase 3	MGI:1921164	5.5	1.1	1.0	0.5	0.8
Ikbke	inhibitor of kappaB kinase epsilon	MGI:1929612	8.7	1.1	0.9	0.9	0.8
Inpp5d/Ship1	inositol polyphosphate-5-phosphatase D	MGI:107357	0.4	0.7	1.0	0.8	1.3
Signal transduction							
Cish	cytokine inducible SH2-containing protein	MGI:103159	38.5	1.5	0.7	0.8	0.7
Socs1	suppressor of cytokine signaling 1	MGI:1354910	20.6	1.6	0.6	1.0	0.8
Apoptosis							
Cflar	CASP8 and FADD-like apoptosis regulator	MGI:1336166	7.6	2.0	1.0	1.1	0.8
Stress response							
Hmox1	heme oxygenase (decycling) 1	MGI:96163	0.7	3.0	1.2	9.4	2.6
Bach1	BTB and CNC homology 1	MGI:894680	0.8	1.4	1.0	1.8	1.1
Cell cycle - transcription factor							
E2f2	E2F transcription factor 2	MGI:1096341	0.3	1.4	0.8	1.0	1.3
Regulation of transcription							
Nfe2l2/Nrf2	nuclear factor, erythroid derived 2, like 2	MGI:108420	1.9	0.9	1.1	0.9	0.9
Cebpb	CCAAT/enhancer binding protein (C/EBP), beta	MGI:88373	3.1	1.8	1.1	1.8	1.2
Etv3	ets variant gene 3	MGI:1350926	3.0	1.1	1.1	1.2	0.9
Spi1	spleen focus forming virus (SFFV) proviral integration oncogene	MGI:98282	2.7	0.9	1.0	0.9	0.8
Maff	v-maf musculoaponeurotic fibrosarcoma oncogene family, protein F (avian)	MGI:96910	2.3	1.2	1.0	1.1	1.0
Trp53inp1	transformation related protein 53 inducible nuclear protein 1	MGI:1926609	1.1	2.2	0.9	1.1	1.2

Mafb	v-maf musculoaponeurotic fibrosarcoma oncogene family, protein B (avian)	MGI:104555	1.0	0.5	0.9	0.2	0.2
Rora	RAR-related orphan receptor alpha	MGI:104661	0.8	2.5	0.9	2.9	1.4
Zfp652	zinc finger protein 652	MGI:2442221	0.5	1.1	1.0	1.2	1.2
Fos	FBJ osteosarcoma oncogene	MGI:95574	0.5	0.4	0.5	0.2	0.8
Nfatc2	nuclear factor of activated T cells, cytoplasmic, calcineurin dependent 2	MGI:102463	0.3	0.7	1.0	0.7	1.3
Notch signaling							
Notch2	notch 2	MGI:97364	2.0	1.0	1.1	1.2	0.9
Tspan14	tetraspanin 14	MGI:1196325	0.5	0.8	1.0	0.8	1.0
Lipid signaling and metabolism							
Lpin1	lipin 1	MGI:1891340	0.1	2.3	0.8	3.0	2.0
S1pr1	sphingosine-1-phosphate receptor 1	MGI:1096355	0.3	1.5	1.2	1.0	1.3
TOR signaling							
Rictor	RPTOR independent companion of MTOR, complex 2	MGI:1926007	2.4	1.0	1.1	0.9	0.8
Membrane transport and secretion							
Slc30a1	solute carrier family 30 (zinc transporter), member 1	MGI:1345281	0.8	3.3	1.2	5.0	1.8
Chrna2	cholinergic receptor, nicotinic, alpha polypeptide 2 (neuronal)	MGI:87886	3.1	0.5	1.3	0.5	0.9
Adhesion and migration							
Ptprj	protein tyrosine phosphatase, receptor type, J	MGI:104574	6.6	0.7	1.3	0.9	0.8
Metabolic							
Cpd	carboxypeptidase D	MGI:107265	4.5	0.9	1.1	0.7	0.9
Agtrap	angiotensin II, type I receptor-associated protein	MGI:1339977	2.0	1.4	0.9	1.0	1.0
Purine and pyrimidine metabolism							
Aicda/Aid	activation-induced cytidine deaminase	MGI:1342279	0.2	1.6	0.8	0.5	1.0

* Accession number available at Mouse Genome Informatics (MGI): http://www.informatics.jax.org/
