# Supplementary material for: miRNA expression profiles and molecular networks in resting and LPS-activated BV-2 microglia—Effect of cannabinoids
Source: PLoS One. 2019 Feb 11;14(2):e0212039. doi: 10.1371/journal.pone.0212039 (PMC6370221; doi:10.1371/journal.pone.0212039)
Supplement: S3 Table — (PDF) [file pone.0212039.s003.pdf]

S3 Table. Selected miR-34a target genes

GeneName	Description	Accession*	Fold change (versus control)			Fold change (versus LPS)	
			LPS	CBD	THC	CBD + LPS	THC + LPS
Immune response and inflammation							
Il6	interleukin 6	MGI:96559	158.8	3.1	0.3	0.5	0.5
Tnf	tumor necrosis factor	MGI:104798	41.1	0.9	1.1	0.7	0.7
Signal transduction							
Il6st	interleukin 6 signal transducer	MGI:96560	0.4	1.0	1.1	1.2	1.2
Axl	AXL receptor tyrosine kinase	MGI:1347244	1.4	3.6	0.5	1.1	1.1
Stress response							
Ddit4	DNA-damage-inducible transcript 4	MGI:1921997	0.5	13.2	1.0	22.8	2.6
Ndrg1	N-myc downstream regulated gene 1	MGI:1341799	0.6	9.6	0.8	5.5	2.3
Slc7a11/xCT	solute carrier family 7 (cationic amino acid transporter, y+ system), member 11	MGI:1347355	1.6	1.4	1.0	2.0	1.1
Notch signaling							
Notch1	notch 1	MGI:97363	6.6	1.3	1.1	1.0	1.1
Notch2	notch 2	MGI:97364	2.0	1.0	1.1	1.2	0.9
Dll1	delta-like 1 (Drosophila)	MGI:104659	60.6	11.2	1.9	0.8	0.9
Cell cycle							
Ccnd1	cyclin D1	MGI:88313	0.3	0.5	0.9	0.4	1.2
Ccne2	cyclin E2	MGI:1329034	0.6	0.5	0.9	0.7	1.0
Cdkn1a/p21	cyclin-dependent kinase inhibitor 1A (P21)	MGI:104556	7.4	3.3	0.6	1.5	0.8
Cdc25a	cell division cycle 25A	MGI:103198	0.8	0.6	0.8	0.6	1.0
E2f5	E2F transcription factor 5	MGI:105091	0.9	0.5	1.2	0.8	1.0
Interferon regulated							
Ifnb1	interferon beta 1, fibroblast	MGI:107657	76.0	4.3	0.4	0.4	0.4
Proinflammatory chemokines							
Ccl22	chemokine (C-C motif) ligand 22	MGI:1306779	10.1	0.9	0.6	0.1	0.6

Lipid signaling and metabolism							
Lpin1	lipin 1	MGI:1891340	0.1	2.3	0.8	3.0	2.0
TGF-b signaling							
Tgif2	TGFB-induced factor homeobox 2	MGI:1915299	0.5	0.8	0.9	0.7	1.1
Regulation of transcription							
Foxp1	forkhead box P1	MGI:1914004	2.2	0.8	1.1	0.8	0.8
Trp53inp1	transformation related protein 53 inducible nuclear protein 1	MGI:1926609	1.1	2.2	0.9	1.1	1.2
Hes2	hairy and enhancer of split 2 (Drosophila)	MGI:1098624	0.7	1.8	1.4	0.4	1.0
Rela	v-rel reticuloendotheliosis viral oncogene homolog A (avian)	MGI:103290	1.7	1.3	1.0	1.4	1.0
Rora	RAR-related orphan receptor alpha	MGI:104661	0.8	2.5	0.9	2.9	1.4
Apoptosis							
Gas1	growth arrest specific 1	MGI:95655	2.8	1.4	1.6	2.0	2.6
Mdm2	transformed mouse 3T3 cell double minute 2	MGI:96952	2.1	1.5	1.0	1.8	1.0
Pdgfrb	platelet derived growth factor receptor, beta polypeptide	MGI:97531	2.4	1.2	0.8	0.8	0.8
Src	Rous sarcoma oncogene	MGI:98397	5.3	1.5	1.0	1.2	0.9
NF-kappaB signaling							
Nfkb1	nuclear factor of kappa light polypeptide gene enhancer in B cells 1, p105	MGI:97312	5.3	1.0	1.0	0.9	0.8
Membrane transport and secretion							
Cnnm3	cyclin M3	MGI:2151055	0.5	1.1	1.1	1.2	1.2
Aqp9	aquaporin 9	MGI:1891066	1.6	9.9	0.7	9.6	1.6
Senescence							
Hbp1	high mobility group box transcription factor 1	MGI:894659	1.9	1.7	1.1	1.3	1.0
Wnt signaling							
Wnt1	wingless-related MMTV integration site 1	MGI:98953	0.9	1.2	0.9	0.6	0.7

* Accession number available at Mouse Genome Informatics (MGI): http://www.informatics.jax.org/
